# P-666. Impact of Ceftriaxone Duration in Community-Acquired Pneumonia: A Retrospective Comparative Analysis

**DOI:** 10.1093/ofid/ofae631.863

**Published:** 2025-01-29

**Authors:** Lyle Ureta, Kevin Aprelian, Hemi Jung, Theresa Lin, Keitaro Kawaguchi, Delia Duenas Hernandez, Romic Eskandarian, Su Lee

**Affiliations:** Adventist Health Glendale, Glendale, California; West Coast University School of Pharmacy, Los Angeles, California; Adventist Health Glendale, Glendale, California; Adventist Health Glendale, Glendale, California; Adventist Health Glendale, Glendale, California; Adventist Health Glendale, Glendale, California; Adventist Health Glendale, Glendale, California; West Coast University School of Pharmacy, Los Angeles, California

## Abstract

**Background:**

In accordance with the American Thoracic Society (ATS) guidelines for community-acquired pneumonia (CAP), ceftriaxone plus a macrolide is commonly used as first-line for inpatient treatment of non-severe CAP(Metlay, 2019). In a community hospital, a CAP protocol was implemented to grant pharmacists the capability to discontinue antibiotics, including ceftriaxone, after 5 days of treatment in hospitalized patients that have reached clinical stability. On the other hand, extended treatment duration of ceftriaxone can lead to increased resistance, *Clostridium difficile* infections, adverse effects and cost (Guo 2017, Thomas 2002). The objective of this study is to compare patient outcomes between patients using ≤5 days (recommended duration) and >5 days (extended duration) of ceftriaxone.

**Methods:**

This study is a retrospective chart review in a community hospital, from Nov 1, 2022 to April 30, 2023. Adult patients treated with ceftriaxone for ≥ 3 days for CAP were included. Patients treated for non-pulmonary infections and/or co-infections were excluded. The primary outcome of this study is 30-day mortality; secondary outcomes include length of stay, and *Clostridium difficile i*nfection within 90 days. Outcomes were analyzed with Chi-square/Fischer’s exact test, normality test, student t-test, and Mann-Whitney U test using Prism Version 10. **IRB Number: 2024-167 AHGL**

**Results:**

The 30-day all-cause mortality was 7.1% for the ≤5 days group (N = 379) and 7.8% for the >5 days group (N = 166) was not statistically significant (p = 0.77). Length of stay median with interquartile range (IQR) was 6 (IQR 4-9) for the ≤5 days group and 8 (IQR 6-11) for the >5 days group (p < 0.01). The Clostridium difficile infection prevalence of 0.5% in the ≤5 days group and 0% in the >5 days group was too low to make any relevant analysis.

**Conclusion:**

This study comparing patient outcomes between ≤5 days and >5 days durations of ceftriaxone for community-acquired pneumonia (CAP) revealed no significant difference in 30-day all-cause mortality rates but a significantly shorter hospital stays in ≤5 days group, which suggests that shorter ceftriaxone durations as per protocol can potentially optimize patient care by reducing hospital stays without compromising mortality outcome.

**Disclosures:**

**All Authors**: No reported disclosures

